# Validity, Reliability, and Sensitivity of Mobile Applications to Assess Change of Direction Speed

**DOI:** 10.5114/jhk/167465

**Published:** 2023-07-15

**Authors:** Hüseyin Şahin Uysal, Alex Ojeda-Aravena, Mehmet Ulaş, Eduardo Báez-San Martín, Rodrigo Ramirez-Campillo

**Affiliations:** 1 Department of Physical Education and Sports Education, Faculty of Sports Sciences, Burdur Mehmet Akif Ersoy University, Burdur, Turkey.; 2 Physical Education School, Pontificia Universidad Católica de Valparaíso, Valparaíso, Chile.; 3 Escuela de Educación, Carrera de Entrenador Deportivo, Universidad Viña del Mar, Viña del Mar, Chile.; 4 Department of Sports Science, Universidad de Playa Ancha, Valparaíso, Chile.; 5 Exercise and Rehabilitation Sciences Institute, School of Physical Therapy, Faculty of Rehabilitation Sciences, Universidad Andres Bello, Santiago, Chile.

**Keywords:** athletic performance, physical fitness, athletes, conditioning, sports

## Abstract

This study aimed to assess the validity, reliability, and sensitivity of mobile applications for assessing change-of-direction speed (CODS) performance. Thirty college athletes performed two Illinois CODS tests during one session. Assessments were carried out simultaneously using six devices (the CODTimer app, Seconds Count app, StopwatchCamera app, two analog stopwatches, and timing gates). Validity analyses included Pearson's product-moment correlation analysis, a linear regression model, and Bland-Altman plots. Reliability analyses included the intraclass correlation coefficient (ICC), the coefficient of variation (CV%), and the paired-sample t test. Sensitivity analyses included the typical error and smallest worthwhile change (SWC). The results showed that validity, reliability, and sensitivity values were higher for the CODTimer app (r = 0.99, R^2^ = 0.99, mean bias = −0.03 ± 0.10, CV% = 3.21, ICC = 0.89, SWC rating: good, p = 0.84) and the Seconds Count app (r = 0.99, R^2^ = 0.99, mean bias = −0.03 ± 0.08, CV% = 3.28, ICC = 0.88, SWC rating: good, p = 0.84) relative to the StopwatchCamera app (r = 0.98, R^2^ = 0.97, mean bias = −0.11 ± 0.22, CV% = 3.43, ICC = 0.86, SWC rating: marginal, p = 0.10), Analog Stopwatch 1 (r = 0.98, R^2^ = 0.96, mean bias = −0.09 ± 0.42, CV% = 2.95, ICC = 0.90, SWC rating: good, p = 0.91), and Analog Stopwatch 2 (r = 0.99, R^2^ = 0.97, mean bias = −0.12 ± 0.88, CV% = 3.51, ICC = 0.87, SWC rating: marginal, p = 0.96). In conclusion, compared to timing gates, the CODTimer app and Seconds Count app provided lower measurement bias and higher sensitivity for assessing CODS performance.

## Introduction

Change-of-direction speed (CODS) is the ability to implement a preplanned change of direction as fast as possible without reacting to a stimulus (Young et al., 2021). CODS is common in sports-related tasks, such as dribbling, shooting and passing (Železnik et al., 2022). An athlete may change activities every 2 to 4 s during a competition and perform 500 to 3000 CODS actions (Taylor et al., 2017). In team sports such as soccer and basketball, an average of 800 CODS movements and 450 lateral movements are performed during a game (Taylor et al., 2017). Furthermore, this physical ability is positively correlated with strength characteristics (i.e., the stretch-shortening cycle, power, maximal dynamic strength, and eccentric accentuated strength), sprint speed, and vertical jump performance (Brughelli et al., 2008; Fernandez-Fernandez et al., 2022; Horníková and Zemková, 2021). In this sense, in college soccer players (n = 44), CODS was positively correlated with 5-m (r = 0.86), 10-m (r = 0.75), and 15-m sprint speed performance (r = 0.55) (Condello et al., 2013). Similarly, in university students (n = 23), there was a moderate positive correlation between CODS (on the lateral shuffle test) and performance on the lateral jump test (r = 0.54) (Mccormick et al., 2014). Additionally, CODS training can improve fitness components that require power, sprint speed, and vertical jumps (Afonso et al., 2020; Condello et al., 2013; Mccormick et al., 2014; Sporis et al., 2010). For example, ten weeks of CODS training improved vertical jump and linear sprint speed performance in healthy college students (n = 80) (Sporis et al., 2010). In addition, CODS performance can affect the athlete's physical condition, regardless of the level of competition, training experience and age (Afonso et al., 2020; Condello et al., 2013; Mccormick et al., 2014; Sporis et al., 2010).

Thus, valid and reliable tests and technologies are required to assess CODS performance (Silva et al., 2021; Willberg et al., 2022). Researchers have developed various devices in an attempt to establish a gold standard. First, CODS performance was assessed with analog stopwatches; later, automatic machines such as timing gates, video timers, and radar systems with high resolution (within ± 0.0005 s) were adopted (Haugen and Buchheit, 2016). Currently, simple and cost-effective mobile applications (apps) that do not require specialized knowledge have also been developed to assess the height of the vertical jump (Bogataj et al., 2020), bar speed (Martínez-Cava et al., 2020) during resistance training, and linear sprint speed-time during running (Peart et al., 2019). In particular, the CODTimer app is used for CODS performance assessment and has established validity/reliability (Balsalobre-Fernández et al., 2019). The gold standard device (i.e., timing gates) and the CODTimer app have a strong positive correlation for 5+5 CODS test performance (r = 0.96, 95% confidence interval [95%CI] = 0.95 to 1.00, standard error of estimate [SEE] = 0.03 s, *p* < 0.001) (Balsalobre-Fernández et al., 2019). Similarly, another study found a strong positive correlation between timing gates and the CODTimer app for the 505 CODS test (r = 0.97, SEE = 0.035 s, *p* < 0.05) (Chen et al., 2021). However, studies have yet to determine the validity, reliability, and sensitivity of current mobile apps, such as the CODTimer app, Seconds Count app, and StopwatchCamera app.

Determining the validity, reliability, and sensitivity of new mobile apps may help coaches identify various options for assessing and monitoring CODS performance. Accordingly, in light of previous evidence (Balsalobre-Fernández et al., 2019; Chen et al., 2021), this study aimed to assess the validity, reliability, and sensitivity of mobile apps for CODS performance. We hypothesized that the CODTimer app, Seconds Count app, and StopwatchCamera app would exhibit suitable validity, reliability, and sensitivity compared with the use of timing gates to assess CODS performance.

## Methods

### Participants

Thirty college athletes (age: 20.3 ± 2.6 years, body height: 174.5 ± 8.1 cm, body mass: 67.5 ± 10.0 kg, body mass index: 22.2 ± 2.6 kg∙m^2^) from the Faculty of Sport Sciences of the Burdur Mehmet Akif Ersoy University participated in this study (for details, see Table 1). Participants consisted of fifteen soccer players (male = 15, female = 0), six basketball players (male = 3, female = 3), four volleyball players (male = 1, female = 3), and five handball players (male = 4, female = 1). The following inclusion criteria were applied: (i) at least one year of training experience, (ii) no injuries in the last six months, (iii) no health problems, (iv) being a team athlete (competing in team sports such as soccer, handball, basketball, or volleyball), and (v) aged between 18–25 years.

**Table 1:** Descriptive characteristics of participants in the study (n = 30).

	Male (n = 23)	Female (n = 7)
Age (y)	20.0 ± 2.0 (18.0 to 23.0)	20.0 ± 1.4 (19.0 to 23.0)
Body mass (kg)	70.0 ± 9.6 (55.0 to 97.0)	59.2 ± 7.8 (45.0 to 68.0)
Body height (cm)	177.4 ± 6.4 (169.0 to 195.0)	164.0 ± 3.5 (160.0 to 171.0)
Body mass index (kg∙m^−2^)	22.1 ± 2.5 (17.3 to 27.6)	22.0 ± 3.2 (16.5 to 26.5)
Training experience (y)	4.4 ± 3.3 (1.0 to 14.0)	1.8 ± 1.8 (1.0 to 6.0)

Note: values are mean ± standard deviation (lower to upper value 95%CI).

Participants included in the study were previously determined according to the simple random method. A minimum sample size of 17 participants was determined from *a priori* analysis (G*Power software, version 3.1, University of Dusseldorf, Germany) using the following settings: bivariate normal model test, two-tailed, α = 0.05, β = 0.90, and r = 0.70 (according to previous studies; Balsalobre-Fernández et al., 2019, Chen et al., 2021). The protocol was approved by the Ethics Committee of the Faculty of Sport Sciences, Mehmet Akif Ersoy University, Burdur, Turkey (Code: 2022/693). The procedures were conducted under the principles of the Declaration of Helsinki (General Assembly of the World Medical Association, 2014).

### Measures

#### Timing Gates

Two pairs of timing gates (Newest Powertimer 300, Finland, 1,000 Hz accuracy) were used as the gold standard to assess the Illinois Agility Test CODS performance (Enoksen et al., 2009). The timing gates were placed in parallel at the first and last stages of the CODS test at a distance of two meters. The height of the timing gate was set at approximately 0.9 m from the ground, corresponding to the height of the participants' hips, to prevent the timing gate from being activated prematurely by a swing of an arm or a leg. After each trial was completed, the total time was recorded manually, and then incorporated in an Excel sheet.

#### CODTimer app

The CODTimer app was developed on the macOS operating system using Xcode 10.2.1 and Swift 5 programming languages. AVFoundation and AVKit frameworks (Apple Inc., USA) supported the mobile app to use slow-motion video features (Balsalobre-Fernández et al., 2019). The CODTimer app was installed on an iPhone 11 (Apple Inc., USA). This CODS mobile app was developed for smartphones with 1920 × 1080 pixel cameras and a recording rate of 240 frames per second (fps) and is compatible with CODS tests (Balsalobre-Fernández et al., 2019; Chen et al., 2021). The assessments with the CODTimer app were taken at a height of one meter, at two meters from the starting line. The start and the end of each trial were considered the first frame in which participants crossed the timing gates with their hips. After the start and end points of the trials were determined, the total time was recorded on the mobile app, and then incorporated in Excel sheets. Data from two Illinois Agility Test CODS trials were included in the analysis.

#### StopwatchCamera app

The StopwatchCamera app was installed on an iPhone 11 (Apple Inc., USA). This mobile app provides video recording at 1280 × 720-pixel quality. The measurements were made manually by video recording. Since there is no slow-motion feature in the StopwatchCamera app, the measurements were carried out depending on the visual ability of independent researchers. Independent researchers used their thumbs to start and end the experiments by pressing the video record button. Similar distance and height values as those used with the CODTimer app were applied (StopwatchCamera app, 2017). After the start and end points of the trials were determined, the total time was recorded manually on the papers created for the study protocol. Then these data were transferred to the electronic environment.

#### Seconds Count app

The Seconds Count app developed by Mensh Technologies was installed on an iPhone 11 (Apple Inc., USA). This mobile app can record videos with different pixel qualities and different frequency values and determine the duration of the athletes' test. The video recording for this study was set at a pixel resolution of 720 HD with a recording rate of 30 frames per second. The assessments were carried out at a height of one meter, at two meters from the timing gates. The test performance was determined by video recording. Video recordings were initiated and stopped as soon as the athlete passed the timing gates (Mensh Technologies, 2018). After the start and end points of the trials were determined, the total time was recorded manually on the papers created for the study protocol. Then these data were transferred to the electronic environment.

#### Analog Stopwatches

Two analog stopwatches (Selex Slx 7064) were used to register the Illinois Agility Test CODS performance (Hetzler et al., 2008). The timing started as soon as the participant started the Illinois Agility Test. Similarly, the stopwatches were stopped when the participant crossed the finish line. Independent researchers were asked to use analog stopwatches with their thumbs while recording the trial periods, and independent researchers were not allowed to communicate with each other throughout the trials. After the start and end points of the trials were determined, the total time was recorded manually on the papers created for the study protocol. Vicente-Rodríguez et al. (2011) argued that the use of analog stopwatches by experienced trainers was valid and reliable for measuring CODS performance.

#### Change-of-Direction Speed Performance

**Figure 1:**
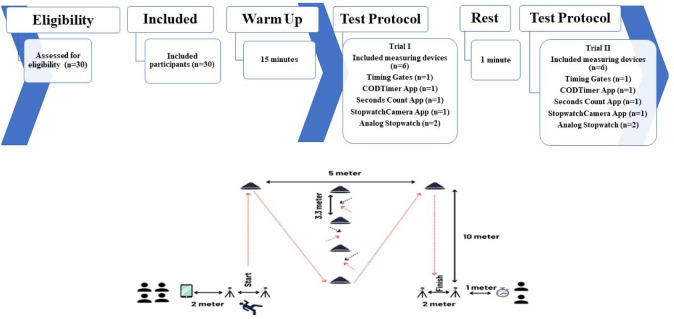
Study flow diagram and change of direction speed (Illinois test) assessment protocol.

CODS performance was assessed using the Illinois Agility Test, in accordance with its widespread use in team sports based on standardized procedures (Hachana et al., 2013; Raya et al., 2013). Briefly, the Illinois CODS test course was marked with four center cones separated by 3.3 meters, and four cones 2.5 meters from the central cones (Raya et al., 2013). The participant started the test lying prone on the floor behind the starting line with his arms at his side, and his head turned to the side or facing forward. On the 'go' command, the participant stood and ran or moved quickly to the first mark on the tape. Participants were required to touch or cross the tape mark with their feet. Then, the participant turned around and moved back to the first center cone, where he wove up and back through the four center cones. The participant then ran or moved as quickly as possible to the second tape mark on the far line. Again, participants must have touched or crossed the final-line tape marks with their foot. Finally, the participant turned around and ran or moved as quickly as possible across the finish line. The time needed to complete each test was recorded and analyzed in seconds and milliseconds (for details, see Figure 1).

#### Design and Procedures

This study used a correlational and predictive design to determine the validity, reliability, and sensitivity of CODS performance through the Illinois Agility Tests using six simultaneous timing methods. *A priori*, the protocol was registered in the Clinicaltrials.gov database (ID: NCT05474521). The testing protocol took place during one day between 09.30 a.m. to 12.00 p.m. in the indoor sports hall of the Faculty of Sports Sciences, Burdur Mehmet Akif Ersoy University and included two timing gates, three smartphone apps, and two analog stopwatches (Analog Stopwatch 1 and Analog Stopwatch 2). The testing protocol was conducted at an ambient temperature of 21°C and 55–60%, respectively, with sufficient sunlight. Only participants and researchers were allowed in the indoor sports hall during the experiments. After the testing equipment was checked (e.g., calibration of timing gates, batteries charge level, etc.), and the Illinois Agility Test course was prepared, a 15-min specific warm-up was performed by participants.

The Illinois Agility Test total time was obtained at 2 m from the start and final points. To quantify the total time for the CODTimer app and Seconds Count app, a video recording was carried out with each device (iPhone 11) at heights of 1.5 m and 2 m from the timing gates. In addition, the StopwatchCamera app recording was made at 1 m from the timing gates. At the same time, two analog stopwatches measured CODS performance at 1 m from the final timing gates. Assistants manually started analog stopwatches when athletes started the test and stopped them once athletes crossed the finish line based on their visual observations. Two Illinois Agility Test trials were administered with one minute of recovery between each test. Details of the procedures can be found at https://www.youtube.com/watch?v=IyrDnICU06I (accessed on 2 June 2022). To reduce interference from uncontrolled variables, all athletes were asked to maintain their regular dietary habits and lifestyle before and during the testing day. However, they were instructed not to exercise the day before the session and to avoid additional high-intensity strength or resistance training sessions, as well as alcohol and caffeine intake, during the experimental period.

#### Statistical Analysis

Data are expressed as the mean ± standard deviation with a 95% confidence interval (95%CI). Data were exported to Microsoft Excel (version 16.37). Statistical processing was performed with SPSS 22.0 (IBM SPSS Statistics, Inc., New York, NY, USA), R version 4.2.2 (Core Team), MedCalc Statistical Software version 19.6.1 (MedCalc Software Ltd), and an Excel spreadsheet created by Hopkins for calculating the typical error (TE) and smallest worthwhile change (SWC) (Hopkins, 2007). The symmetry and normality assumptions were verified by Skewness-Kurtosis and the Shapiro-Wilk test, respectively (George and Mallery, 2010). The content validity between the CODS measurements of the timing gates and devices was determined by an independent-sample *t* test. Concurrent validity was determined by the Pearson's product-moment correlation coefficient (r) and by a simple linear regression (Hopkins et al., 2009). The correlation coefficient was interpreted according to the following reference values: trivial (< 0.10), small (0.10–0.29), moderate (0.30–0.49), large (0.50–0.69), very large (0.70–0.89), or excellent (> 0.90) (Hopkins et al., 2009). To meet linear regression assumptions, the residuals standardized were analyzed to detect outliers (Maloney et al., 2017). The possibility of collinearity between the predictor variables was examined using the variance inflation factor (VIF; VIF < 10) and tolerance (> 0.2), and verified by the Durbin-Watson test. Additionally, the presence of systematic and proportional bias between each measurement was examined visually using Bland-Altman plots (Atkinson and Nevill, 1998). Relative intrasession test-retest reliability was determined by the intraclass correlation coefficient (ICC), and absolute reliability was determined by the coefficient of variation (CV%). The following values were used to interpret the ICC (Koo and Li, 2016): poor (> 0.50), moderate (> 0.50–0.75), good (0.75–0.90), and excellent (0.90–1). In addition, if the CV was less than 10%, the test-retest validity was considered acceptable (Buchheit et al., 2011). Consistency between the CODS measurements of the timing gates and devices was assessed by an independent-sample *t* test (Chen et al., 2021). Differences between measurements were determined by the effect size (ES) measured in terms of Cohen’s *d* (Cohen, 2013):


$$Cohen's\;d=\frac{M_{1}-M_{2}}{SD_{pooled}},SD_{pooled}=\frac{\sqrt{SD_{1}^{2}-SD_{2}^{2}}}{2}$$

Effect size was interpreted according to the following reference values: trivial (<0.2), small (0.60–1.19), large (1.2–1.99) and very large (>2) (Cohen, 2013). The sensitivity of technological devices was analyzed in terms of the smallest worthwhile change and typical error (Hopkins, 2004). The typical error (TE) was a measure of variation between two trials used in test-retest reliability analyses (Hopkins, 2007). When the typical error was less than the smallest worthwhile change, the evaluated test (or test device) was considered sensitive (Nascimento et al., 2017). These values were interpreted as follows: marginal (TE > SWC), satisfactory (TE = SWC), or good (TE < SWC) (Hopkins, 2007). The alpha value was set at 0.05.

## Results

### Content and Concurrent Validity

All mobile apps showed similar internal consistency and means (*p* > 0.05). Specifically, small differences were reported in the validity values of the timing gates (*d* = 0.25, trivial), CODTimer app (*d* = 0.21, small), Seconds Count app (*d* = 0.21, small), StopwatchCamera app (*d* = 0.21, small), Analog Stopwatch 1 (*d* = 0.23, small), and Analog Stopwatch 2 (*d* = 0.24) (Table 2).

**Table 2:** Mean difference and consistency of measurement for change-of-direction speed time.

	Trial 1 Mean±SD	Trial 2 Mean±SD	*p*-value*	ES	ES rating	Mean difference (95%CI)
Timing gates	18.65 ± 1.4	18.64 ± 1.1	0.98	0.25	Small	0.00 (−0.66 to 0.68)
CODTimer app	18.70 ± 1.4	18.64 ± 1.1	0.84	0.21	Small	0.06 (−0.60 to 0.73)
Seconds Count app	18.71 ± 1.4	18.64 ± 1.1	0.84	0.21	Small	0.06 (−0.60 to 0.73)
StopwatchCamera app	18.73 ± 1.3	18.79 ± 1.1	0.10	0.21	Small	−0.06 (−0.71 to 0.59)
Analog Stopwatch 1	18.72 ± 1.3	18.76 ± 1.2	0.91	0.23	Small	−0.03 (−0.71 to 0.64)
Analog Stopwatch 2	18.76 ± 1.2	18.77 ± 1.4	0.96	0.24	Small	−0.01 (−0.72 to 0.68)

SD: standard deviation; *: paired sample t-test; ES: effect size (Cohen’s d); CI: confidence interval.

In addition, the six mobile apps indicated concurrent validity. Specifically, perfect correlations were reported between the timing gates and the CODTimer app (r = 0.99, [95%CI = 0.99 to 0.99], *p* < 0.05) and the Seconds Count app (r = 0.99, [95%CI = 0.99 to 0.99], *p* < 0.05). Very large correlations were reported between the timing gates and Analog Stopwatch 2 (r = 0.99, [95%CI = 0.98 to 0.99], *p* < 0.05), Analog Stopwatch 1 (r = 0.98, [95%CI = 0.97 to 0.99], *p* < 0.05), and the StopwatchCamera app (r = 0.98, [95%CI = 0.97 to 0.99], *p* < 0.05). Additionally, the linear regression model showed that all devices predicted CODS performance including the CODTimer app (R^2^ = 0.99, β = 0.96, *p* = 0.00), Seconds Count app (R^2^ = 0.99, β = 0.97, *p* = 0.00), StopwatchCamera app (R^2^ = 0.97, β = 0.95, *p* = 0.00), Analog Stopwatch 1 (R^2^ = 0.96, β = 0.98, *p* = 0.00), and Analog Stopwatch 2 (R^2^ = 0.97, β = 0.99, *p* = 0.00). Correlations and linear regression details are presented in Table 3.

**Table 3:** Correlation and regression values between mobile apps and timing gates.

	r (lower to upper 95%CI)	Magnitude	Equation	R^2^ adjusted	B
CODTimer app	0.99 (0.99 to 0.99)*	Perfect	Y = 0.9695*X + 0.5837	0.99	0.96*
Seconds Count app	0.99 (0.99 to 0.99)*	Perfect	Y = 0.9730*X + 0.5237	0.99	0.97*
StopwatchCamera app	0.98 (0.97 to 0.99)*	Very large	Y = 0.9591*X + 0.8351	0.97	0.95*
Analog Stopwatch 1	0.98 (0.97 to 0.99)*	Very large	Y = 0.9824*X + 0.4605	0.96	0.98*
Analog Stopwatch 2	0.99 (0.98 to 0.99)*	Perfect	Y = 0.9997*X + 0.1010	0.97	0.99*

* Denotes p < 0.001; CI: confidence interval; r: Pearson correlation coefficient; Magnitude: Pearson correlation coefficient effect size; B: Beta coefficient.

### Reliability

We found good to excellent levels of relative intrasession reliability in the timing gates (ICC = 0.88, [95%CI = 0.74 to 0.94]), CODTimer app (ICC = 0.89, [95%CI = 0.76 to 0.95]), Seconds Count app (ICC = 0.88, [95%CI = 0.74 to 0.94]), StopwatchCamera app (ICC = 0.86, [95%CI = 0.70 to 0.93]), Analog Stopwatch 1 (ICC = 0.90, [95%CI = 0.80 to 0.97]), and Analog Stopwatch 2 (ICC = 0.87, [95%CI = 0.72 to 0.94]).

**Figure 2:**
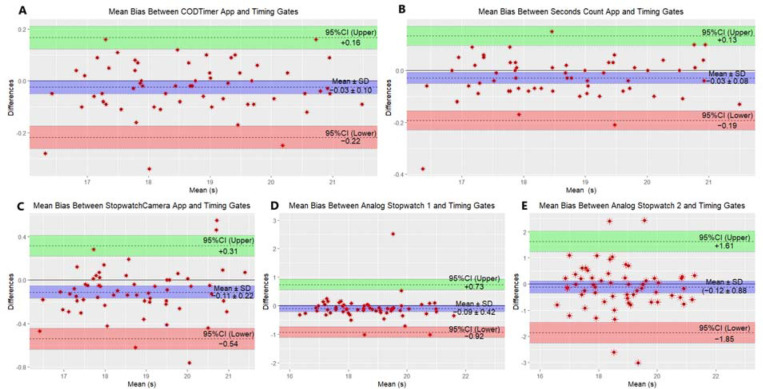
Mean bias between measurement devices. **A:** CODTimer App and timing gates (−0.03 ± 0.10; −0.22 to 0.16); **B:** Seconds Count App and timing gates (−0.03 ± 0.08; −0.19 to 0.13); **C:** Stopwatch Camera App and timing gates (−0.11 ± 0.22; −0.54 to 0.31); **D:** Analog Stopwatch 1 and timing gates (−0.09 ± 0.42; −0.92 to 0.73); **E**: Analog Stopwatch 2 and timing gates (−0.12 ± 0.88; −1.85 to 1.61). Note: Values are mean ± standard deviation (SD) (95% confidence interval[95%CI]). Athletes' best test times were used to create the Bland Altman plots.

Similarly, the data indicated acceptable absolute reliability for the CODTimer app (CV = 3.21%, [95%CI = 2.35 to 4.07]), Seconds Count app (CV = 3.28%, [95%CI = 2.41 to 4.17]), StopwatchCamera app (CV = 3.43%, [95%CI = 2.51 to 4.35]), Analog Stopwatch 1 (CV = 2.95%, [95%CI= 2.16 to 3.74]), and Analog Stopwatch 2 (CV = 3.51%, [95%CI = 2.57 to 4.46]). Differences are shown in the Bland-Altmann plot in Figure 2.

### Sensitivity

The sensitivity of the devices assessed was rated from marginal to good according to the Illinois Agility Test. The timing gates showed satisfactory sensitivity, the CODTimer app showed good sensitivity, the Seconds Count app showed good sensitivity, the StopwatchCamera app showed marginal sensitivity, Analog Stopwatch 1 showed good sensitivity, and Analog Stopwatch 2 showed marginal sensitivity. Table 4 provides detailed information on the TE and SWC values.

**Table 4:** Descriptive statistics on the reliability and usefulness values between technological devices and timing gates.

Variables	ICC (95%CI)	ICC rating	CV (%) (95%CI)	TE (95%CI)	SWC (95%CI)	SWC rating
CODTimer app	0.89 (0.76 to 0.95)	Good	3.21 (2.35 to 4.07)	0.63 (0.48 to 0.93)	0.67 (0.55 to 0.85)	Good
Seconds Count app	0.88 (0.74 to 0.95)	Good	3.28 (2.41 to 4.17)	0.65 (0.49 to 0.96)	0.67 (0.55 to 0.85)	Good
StopwatchCamera app	0.86 (0.70 to 0.94)	Good	3.43 (2.51 to 4.35)	0.68 (0.52 to 1.01)	0.67 (0.55 to 0.86)	Marginal
Analog Stopwatch 1	0.90 (0.80 to 0.96)	Excellent	2.95 (2.16 to 3.74)	0.56 (0.43 to 0.83)	0.67 (0.55 to 0.86)	Good
Analog Stopwatch 2	0.87 (0.72 to 0.94)	Good	3.51 (2.57 to 4.46)	0.69 (0.52 to 1.02)	0.67 (0.55 to 0.86)	Marginal

CV: Coefficient of variation; ICC: Intraclass correlation coefficient; SWC: Smallest worthwhile change (relative values); TE: Typical error; CI: Confidence interval.

## Discussion

This study aimed to assess the validity, reliability, and sensitivity of mobile apps for measuring change-of-direction speed performance. Our hypothesis was partially supported. The CODTimer app and Seconds Count app were valid, reliable, and sensitive methods of assessing CODS performance. On the other hand, mean biases in CODS performance were observed with the StopwatchCamera app. Although the StopwatchCamera app and analog stopwatches were reliable and valid, a significant mean bias and marginal sensitivity of the StopwatchCamera app were observed.

Specifically, the CODTimer app is a valid and reliable mobile app for assessing the 5+5 and 505 CODS tests (Balsalobre-Fernández et al., 2019; Chen et al., 2021). In our study, the Illinois CODS test assessed with the CODTimer app obtained higher validity (r = 0.99, *p* < 0.05) than in previous studies (Balsalobre-Fernández et al., 2019; Chen et al., 2021). This may be due to the location and distance at which smartphones were placed for performance tests. In the 5+5 CODS test, researchers placed their smartphones 2 m away from the timing gates (Balsalobre-Fernández et al., 2019). However, smartphone cameras recorded athletes in motion during all tests (Balsalobre-Fernández et al., 2019). In contrast, smartphones remained stationary during recording of the 505 CODS tests (Chen et al., 2021). Researchers set a distance of 6 m between the gold standard (i.e., timing gates) and smartphone cameras (Chen et al., 2021). The study protocols used to evaluate the CODTimer app varied in terms of athlete groups, iPhone models, temperatures, and times of day. Similar levels of relative reliability to our current findings (ICC = 0.89, *p* < 0.05) were identified in previous studies (Balsalobre-Fernández et al., 2019; Chen et al., 2021). The CODTimer app produced reliable results in the 5+5, 505, and Illinois CODS tests, regardless of the variables measured. Furthermore, the CODTimer app was sensitive, as indicated by the TE and SWC values (TE = 0.63, SWC = 0.67). This study is the first to demonstrate the sensitivity of the CODTimer App.

This study was also the first to examine the validity, reliability, and sensitivity of the Seconds Count app for measuring CODS performance. The Seconds Count app showed high validity, reliability, and sensitivity compared to the timing gates (r = 0.99, *p* < 0.05, ICC = 0.88, TE = 0.65, SWC = 0.67). The reason for these results could be the high-resolution video recording function. The CODTimer app records videos with a resolution of 1920 × 1080 pixels, while Seconds Count app can record videos with a resolution of 720 high definition (HD). Another critical feature that distinguishes between these two mobile apps is the slow motion and video analysis options. Stanton et al. (2017) noted that performance-recording apps with slow-motion features could increase the reliability of results (Stanton et al., 2017). In our study, video recordings were able to detect motion at 240 fps and 30 fps due to the slow-motion functions of the CODTimer app and the Seconds Count app. Furthermore, the CODTimer app produced similar results to the Seconds Count app at 30 fps, despite having a high recording rate of 240 fps. Thus, the CODTimer app was valid, reliable, and sensitive for CODS measurements with a phone camera recording rate of 30 fps.

The other application of which validity, reliability, and sensitivity were examined for the first time in this study was the StopwatchCamera app. The StopwatchCamera app had good reliability and validity (r = 0.98, [95%CI = 0.97 to 0.99, *p* < 0.05, ICC = 0.86, [95%CI = 0.70 to 0.93). However, this mobile app did not have slow-motion or video recording features like the other apps. Therefore, it was considered a marginal device for assessing CODS performance (TE = 0.68, SWC = 0.67).

Additionally, we measured CODS with analog stopwatches. Our results indicated that Analog Stopwatch 1 had good validity, reliability, and sensitivity for assessing CODS performance (r = 0.98, *p* < 0.05, ICC = 0.90, TE = 0.56, SWC = 0.67). However, Analog Stopwatch 2 was worse at assessing CODS performance in terms of these characteristics (r = 0.98, *p* < 0.05, ICC = 0.87, TE = 0.69, SWC = 0.67). Previous studies have also reported similar contradictory results, and analog stopwatches were not considered for measurement due to their low sensitivity and mean error rates (Chen et al., 2021; Hetzler et al., 2008; Mann et al., 2015; Vicente-Rodríguez et al., 2011). Given our findings, analog stopwatches are not an adequate instrument for assessing CODS performance.

Potential limitations of this study should be noted. In the Illinois CODS test, all devices measured the athletes' total test times at values higher than those measured by the timing gates. This may be due to the difference between the test starting point of the timing gates and that of practitioners (Stanton et al., 2017). Stanton et al. (2017) stated that if the test starting points differed, there would be a mean bias between −0.62 and 0.84 in the measurements collected by practitioners compared with the fully automated gold standard test devices. Although the means of measuring devices were higher than those of timing gates, differences in means between the measuring devices were non-significant (*p* < 0.05). On the other hand, the systematic mean bias was minimal in the measurements collected with the CODTimer app and the Seconds Count app (mean bias [CODTimer app = −0.03 ± 0.10], [Seconds Count app = −0.03 ± 0.08]). In contrast, the systematic mean biases of the StopwatchCamera app and the Analog Stopwatch 1 were higher than those of the CODTimer app and the Seconds Count app (mean bias [StopwatchCamera app = −0.11 ± 0.22], [Analog Stopwatch 1 = −0.09 ± 0.42, −0.12 ± 0.88]).

With technological advances, the frequency of use of mobile applications is increasing due to their accessibility and practicality (Peart et al., 2019). Thus, the results of this study are relevant for researchers and coaches in the field. We showed that the mobile apps CODTimer and Seconds Count are valid, reliable, and sensitive for assessing CODS. Alternatively, the StopwatchCamera app and analog stopwatches can be used. However, the scope of this study was limited to the Illinois CODS test. The CODTimer app has been shown to provide valid and reliable results at different times of day and in other performance tests. However, future studies should focus on analyzing how various situations might affect image quality (such as nighttime assessments, foggy weather, and far distances). In addition, although all studies were conducted on iPhones, the validity, reliability, and sensitivity of the CODTimer app on phones with the Android operating system were not assessed. As a result, researchers and practitioners without access to the gold standard devices can use the CODTimer app and Seconds Count app to obtain valid, reliable, and sensitive measurements.

Coaches and practitioners may prefer the CODTimer app and the Seconds Count app over timing gates to evaluate CODS performance. The CODTimer app is a paid app. However, researchers who only want to assess the test time of athletes can access the Seconds Count app for free. Researchers who wish to comprehensively assess CODS performance can purchase the CODTimer app. Measurements should be collected from 2 m behind the start and finish lines to achieve valid, reliable, and sensitive results. Keeping the camera stationary throughout the test was essential to achieve accurate results. To avoid mean bias, focusing on the joint limb of each athlete (for example fingers, head, or knees) or placing markers can improve the quality of the test. The test should be conducted with adequate environmental conditions (e.g., sufficient sunlight), for valid, reliable, and sensitive measurements.

## Conclusions

This study has demonstrated that the CODTimer App and the Seconds Count App are reliable, valid, and sensitive mobile applications for measuring change-of-direction speed (CODS) performance. When utilized with iPhone devices positioned at 2 m, these mobile apps yield results comparable to those of benchmark measurement devices. Consequently, coaches and professionals can confidently employ these applications to assess CODS performance, providing a convenient and accessible alternative to traditional methods
